# The Association between Cardiorespiratory Fitness and Gut Microbiota Composition in Premenopausal Women

**DOI:** 10.3390/nu9080792

**Published:** 2017-07-25

**Authors:** Yifan Yang, Yi Shi, Petri Wiklund, Xiao Tan, Na Wu, Xiaobo Zhang, Olli Tikkanen, Chenhong Zhang, Eveliina Munukka, Sulin Cheng

**Affiliations:** 1 School of Life Sciences and Biotechnology, Shanghai Jiao Tong University, Shanghai 200240, China; yifanyang89@outlook.com (Y.Y.); wuna0308@163.com (N.W.); xbzhang@aliyun.com (X.Z.); lltkknn@gmail.com (O.T.); 2 Exercise, Health and Technology center, Shanghai Jiao Tong University, Shanghai 200240, China; petri.wiklund@jyu.fi (P.W.); xiao.tan@jyu.fi (X.T.); 3 Key Laboratory of Systems Biomedicine, Ministry of Education, Shanghai Centre for Systems Biomedicine, Shanghai Jiao Tong University, Shanghai 200240, China; 4 Faculty of Sport and Health Sciences, University of Jyvaskyla, 40014 Jyvaskyla, Finland; 5 Center for Life Course Epidemiology, Faculty of Medicine, University of Oulu, 90014 Oulu, Finland; 6 Department of Epidemiology and Biostatistics, School of Public Health, Imperial College, SW7 2AZ London, UK; 7 State Key Laboratory of Microbial Metabolism, School of Life Sciences and Biotechnology, Shanghai Jiao Tong University, Shanghai 200240, China; zhangchenhong@sjtu.edu.cn; 8 Department of Medical Microbiology and Immunology, University of Turku, 20014 Turku, Finland; emunukka@gmail.com

**Keywords:** exercise, VO_2max_, gut microbiota, body fatness

## Abstract

The aim of this study was to investigate the association between cardiorespiratory fitness and gut microbiota composition in premenopausal women. The participants consisted of 71 premenopausal Finnish women (aged 19–49 years). Gut microbiota were analyzed using flow cytometry, 16S rRNA gene hybridization and DNA-staining. Maximum oxygen uptake (VO_2max_) was assessed by respiratory gas analyzer and body composition by Bioimpdance. We found that participants with low VO_2max_ had lower *Bacteroides*, but higher *Eubacterium rectale-Clostridium coccoides* than the high VO_2max_ group (*p* < 0.05 for all). VO_2max_ was inversely associated with *EreC* (*r* = −0.309, *p* = 0.01) but not with other bacteria. VO_2max_ also negatively correlated with fat% (*r* = −0.755, *p* < 0.001), triglycerides (*r* = −0.274, *p* = 0.021) and leptin (*r* = −0.574, *p* < 0.001). By contrast, *EreC* was positively associated with fat% (*r* = 0.382, *p* = 0.002), dietary fat intake (*r* = 0.258, *p* = 0.034), triglycerides (*r* = 0.390, *p* = 0.002) and leptin (*r* = 0.424, *p* = 0.001), but negatively with carbohydrate intake (*r* = −0.252, *p* = 0.034) and HDL (*r* = −0.26, *p* = 0.028). After adjusting for age and dietary intake, all the significant associations remained. However, after adjusting for fat%, the associations between VO_2max_ and *EreC* disappeared. Our results suggest that cardiorespiratory fitness is associated with gut microbiota composition, independent of age and carbohydrate or fat intake. The association between VO_2max_ and *EreC*, however, appears to be mediated by body fatness.

## 1. Introduction

There is growing awareness that microbial communities colonize different regions of the gastrointestinal tract, playing a major role in the health and disease of their host [1]. In the healthy state, the commensal microbes help to digest and absorb nutrients, modulate the immune system and provide protection against enteropathogens [1,2]. Gut microbial imbalance, followed by a state of dysbiosis, in turn, is associated with obesity [3], type 2 diabetes [4], cardiovascular disease [5] and non-alcoholic fatty liver disease [6]. Modulation of the gut microbiome has therefore become a topic of considerable interest.

Although using antibiotics is considered to be an efficient way to modify the microbiota, recent studies have suggested that dietary modification and regular exercise may offer cost-effective alternative means to achieve the same end [7,8]. A cross-sectional study of professional rugby players showed that exercise is associated with gut microbial diversity, and that proportions of several microbial taxa were significantly higher in the rugby players compared with the control group [9]. A recent study by Estaki et al. [10] also showed a positive correlation between cardiorespiratory fitness and microbial diversity in a small group of young healthy adults. However, earlier studies were done in athletic men and young adults; a current study has shown that estrogen in women influences gut microbiota [11], but overweight or obese women with relatively low physical fitness remain to be studied.

Physical fitness is considered one of the most objective measures of the level of physical activity [12]. Cardiorespiratory fitness is the overall capacity of the cardiovascular and respiratory systems and the ability to carry out prolonged strenuous exercise. The maximal oxygen consumption (VO_2max_) attained during a graded maximal exercise to voluntary exhaustion has long been considered by the World Health Organization as the single best indicator of cardiorespiratory fitness [13]. Therefore, to study whether the level of physical fitness is associated with certain compositions of the gut microbiota, using VO_2max_ as a segregate variable is possible option in a cross-sectional study design. The aim of the present study was to examine the relationship between gut microbiota and aerobic fitness in premenopausal women with diverse body composition (mostly overweight or obese with a sedentary lifestyle). More specifically, we examined (1) the correlations between cardiorespiratory fitness and specific bacteria groups, and (2) whether these correlations between cardiorespiratory fitness and specific bacteria groups were independent of age, dietary intake and whole-body fat mass.

## 2. Materials and Methods

### 2.1. Study Design and Subjects

The study participants consisted of 71 Finnish women (aged 19 to 49 years) who resided in the city of Jyvaskyla, Central Finland, which has a population of approximately 150,000 [14]. In our early study, 80% of the people who participated in our study were still living in Jyvaskyla after 10 years and there were no differences between people who were living inside the city compared to those who were living outside the city (unpublished data). However, duration of living in the city of Jyvaskyla was not a required inclusion criterion. The majority (80%) of the study participants were either overweight or obese; but had no diagnosed cardiovascular disease, type I or type II diabetes or serious musculoskeletal problems. None of the participants had been on antibiotics treatment during the past three months. The study protocol was approved by the ethical committee of the Central Finland Health Care District (No: 7/2011). Informed consent was obtained from each participant prior to the assessments.

### 2.2. Background Information

Background information, including medical history and current health status, was collected via self-administered questionnaires. The level of physical activity in terms of duration (exercise hours per week) and frequency (exercise times per week) was also collected by validated questionnaires [15,16]. Food consumption and intakes of total energy and energy-yielding nutrients were assessed from food records that were kept for three days (including two weekdays and one weekend day). Food diary records were analyzed for total calorie, protein, carbohydrate, and fat intake by using the software Micro-Nutrica (v3.1), developed by the Social Insurance Institution of Finland and updated with a database for new foodstuffs by the study nutritionist [17]. To minimize possible under-reporting, the proportion of total energy intake of energy-yielding nutrients (E%) was calculated and reported in this study.

### 2.3. Anthropometrical and Body Composition Assessments

Body height (cm) was measured using standardized protocols (a wall-fixed measuring device). Body weight (kg) and whole-body fat mass were assessed with light clothes and without shoes using bio-impedance (Inbody 720, Biospace Co. Ltd., Seoul, South Korea). Body mass index (BMI) was calculated according to weight/height^2^ (kg·m^−2^). Percent of body fat (fat%) was calculated as whole-body fat mass divided by weight.

### 2.4. Fitness Test

The maximum oxygen uptake (VO_2max_ in mL/kg/min) was assessed by a bicycle ergometer under the supervision of a physician. The test began with a 2-min warm-up at 50 W. After that the intensity was increased by 25 W at 2-min intervals until exhaustion. Electrocardiography was monitored continuously and heart rate and maximal work load were recorded at the end of every load. Oxygen uptake was assessed by the breath-by-breath method using a respiratory gas analyzer (Sensor Medics Vmax, Yorba Linda, CA, USA). Maximal oxygen uptake was reached when the measured VO_2_ reached a plateau or started to decrease, or the subject felt she had reached her maximal level and wanted to stop the test. On the basis of the VO_2max_ values, participants were divided into three groups by tertiles (low, moderate and high).

### 2.5. Blood and Biochemicla Measurements

Venous blood samples were taken in standardized fasting condition (12 h) in the morning (7–9 a.m.). Serum samples were stored frozen at −80 °C until analyzed. Serum triglycerides, total cholesterol and high-density lipoprotein (HDL) were determined by using KONELAB 20XTi analyzer (Thermo Fischer Scientific Inc, Waltham, MA, USA) and described previously [18]. The intra- and inter-assay correlation coefficients (CVs%) were 3.4% and 2.9% for triglycerides. Serum leptin was assessed using human leptin (ELISA; Diagnostic Systems Laboratories, Inc., Webster, TX, USA). The inter- and intra-assay coefficients of variation (CVs%) were 2.2% and 2.7% for leptin, respectively.

### 2.6. Fecal Samples

Fecal samples were taken from evacuated stool by the subjects with detailed guidance and frozen immediately and stored at −70 °C until processing. The bacterial cells were separated and analyzed with a previously described method using 16S rRNA hybridization, DNA-staining, and flow cytometry [19,20]. The following five 16S rRNA-targeted oligonucleotide probes labelled at the 5′-end with Cy5 indocarbocyanine (Ex/Em 646/662 nm; Molecular Probes, Eugene, OR, USA) were used: Bacto1080 for *Bacteroides* group, Bif164 for *Bifidobacterium* species, Enter1432 for *Enterobacteria* (Ent), EreC482 for *Eubacterium rectale-Clostridium coccoides* group (*EreC* group) and Fprau645 for *Faecalibacterim prausnitzii* (F.p).

### 2.7. Statistical Analyses

Data analyses were carried out using SPSS Statistics 22 for Windows (SPSS Inc., Chicago, IL, USA). All data was checked for normality using the Shapiro–Wilk W-test. If data were not normally distributed, their natural logarithms were used. Multiple comparisons were applied by using analysis of variance (ANOVA) with the Sidak post-hoc test.

Linear regression was used to assess the relationship between cardio-respiratory fitness and gut microbiota. The analyses were performed with and without adjusting for age, dietary intake and fat%. The *p*-values were adjusted by multiple comparisons in FDR (false discovery rate). Statistical significance was set at *p* < 0.05 with 2-tails.

In addition, multivariable least square regressions were performed in assessing the contribution of the variables (i.e., *EreC*, age, energy yield nutrients of fat, carbohydrates, protein and alcohol, as well as fat% of the whole body) and the outcome variables (i.e., VO_2max_, leptin, HDL and TG). To make each outcome comparable, we standardized each column by means of z-scoring so that each column has mean value 0 and standard deviation 1. The Pearson correlation coefficients between the regressed outcome and the observed outcome were calculated and regression weights for each variable were provided. The higher the absolute weight, the higher the contribution.

## 3. Results

### 3.1. Descriptive Statistics

The physical characteristics of the study participants are given in Table 1. The high aerobic fitness group was relatively younger than the low aerobic fitness and control groups (*p* < 0.01). The high aerobic fitness group weighed less, had lower BMI and fat% and were more active than the other two groups (*p* < 0.005). No significant differences in energy yield nutrients (carbohydrate, protein, fat) and fiber intake were found among the groups. The high aerobic fitness group had lower serum triglycerides and leptin (*p* < 0.05), and tended to have lower cholesterol concentration than the low aerobic fitness group.

### 3.2. Comparison of Microbial Composition in Different Fitness Groups

**Figure 1:**
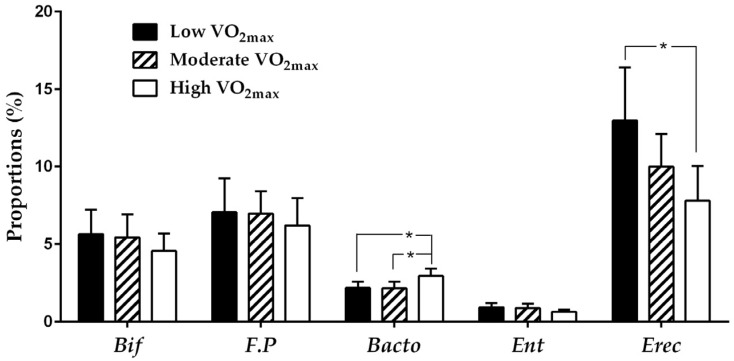
The microbiota composition in different fitness groups. The error bars represent mean and standard deviation. The contrast estimates (*p*-values) by multivariable comparison with Sidak to localize the significant differences between the groups. * means *p* < 0.05. *Bif* = *Bifidobacterium*, *F.p* = *Faecalibacterim prausnitzii*, *Bacto* = *Bacteroides*, *Ent* = *Enterobacteria*, *EreC* = *Eubacterium rectale-Clostridium coccoides*.

The microbiota composition of the different fitness groups are presented in Figure 1. Using multivariable comparison, we found that the high fitness group had higher proportions of *Bacteroides* (*p* = 0.038) but lower *EreC* (*p* = 0.040) than the low fitness group. No significant differences were found in *Bifidobacterium*, *Enterobacteria* and *Faecalibacterium prausnitzii* among the groups.

### 3.3. Associations of Certain Bacterial Groups with Different Variables

**Figure 2:**
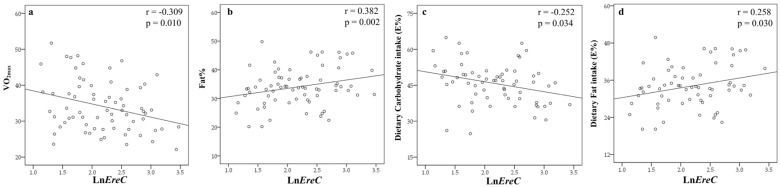
The associations of *EreC* group with VO_2max_, fat%, and energy yield (E%) nutrients. (**a**) EreC was inversely correlated with VO2max; (**b**) EreC was positively correlated with fat%; (**c**) EreC was inversely correlated with carbohydrate intake (E%); (**d**) EreC was positively correlated with fat intake (E%). Ln = log transferred values; *EreC* = *Eubacterium rectale-Clostridium coccoides*.

The associations of certain bacterial groups with VO_2max_, fat%, and energy yield nutrients are presented in Figure 2. We found that *EreC* was inversely correlated with VO_2max_ (*p* = 0.010, Figure 2a) and carbohydrate intake (*p* = 0.034, Figure 2c), but positively with fat% (*p* = 0.002, Figure 2b) and fat intake (*p* = 0.034, Figure 2d). *EreC* was also negatively correlated with HDL (*p* = 0.028), but positively with triglycerides (*p* = 0.002) and leptin (*p* = 0.001, Table 2). No other bacterial groups were correlated with VO_2max_. On the other hand, VO_2max_ was negatively correlated with fat% (*r* = −0.755, *p* < 0.001), triglycerides (*r* = −0.274, *p* = 0.021) and leptin (*r* = −0.574, *p* < 0.001). After adjusting for the confounding factors of age and energy yield of macronutrient intakes (carbohydrate, protein, fat and alcohol), the associations between bacteria and metabolic traits remained (Table 2). However, when fat% was included in the model, the significant differences between *EreC* and VO_2max_, triglycerides and leptin disappeared.

In addition, each variable contribution to the multivariable associations between variables X (i.e., *EreC*, age, energy yield nutrients of fat, carbohydrates, protein and alcohol, as well as fat% of the whole body) and outcomes Y (i.e., VO_2max_, leptin, HDL and TG) are given in the Table 3. The Pearson correlation coefficient between the regressed outcome antable d the observed outcome are 0.838, 0.813, 0.416, and 0.551, respectively, with significant *p* < 0.001 for all. *EreC* contributed the most to the triglycerides followed by HDL, leptin and VO_2max_. Fat% has the highest contribution to VO_2max_.

Further, we performed a multi-variable regression analysis between the five intestinal bacteria (X) and the five outcomes (Y). The contribution weights are listed in Table 4. The Pearson correlation coefficients between the regressed outcome and the observed outcome are 0.350, 0.428, 0.442, 0.431, and 0.477, with a significance of 0.003, <0.001, <0.001, <0.001, and <0.001, respectively. *EreC* has the highest contribution among all the bacteria to the outcomes (VO_2max_, fat%, leptin, HDL and TG, respectively).

## 4. Discussion

In this study, we found that gut microbiota was associated with cardiorespiratory fitness in young and middle-aged women. The study participants with low aerobic fitness had higher *Eubacterium rectale-Clostridium coccoides* and *Enterobacteria*, but lower *Bacteroides*. These differences were independent of age and macronutrient intake, but appeared to be confounded by adiposity, since all differences between the groups disappeared after adjusting for the percent of body fat.

There is growing interest in gut microbiota and their role in health and disease. However, research in this field is still in the beginning phase, therefore, the results are inconclusive but illustrative. It has been shown that obese mice had significantly lower *Bacteroidetes* but a higher proportion of *Firmicutes* compared to lean mice [21]. A similar observation was also found in a human study when comparing obese to lean twins [22]. Previous studies also showed that the *EreC* group is associated with obesity and related to metabolic disorders [23,24,25,26]. Our current results are in agreement with these studies by showing that the low VO_2max_ group (which had a high BMI) had significantly lower *Bacteroides* but higher *EreC* (phylum *Firmicutes*) and *Enterobacteria* (Phylum *Proteobacteria). EreC* contributes the most to the regression of VO_2max_. However, Goodrich et al. found in the large TwinsUK population study that *Bacteroidales* (phylum *Bacteroidetes*) and family *Clostridiaceae* (phylum *Firmicutes*) correlated negatively with BMI and triglycerides [27]. Similar results were reported in another large population-based cohort by Fu et al. [28] The discrepancy between these studies may partly be due to differences in sample size, study population and the methods used to assess the bacteria, it might also be because different bacterial species (within the same phyla) have different functions and thus also different associations. In addition, the intestinal microbiota is known to regulate host energy homeostasis and can be influenced by diet and other environmental factors which are still not well characterized [28].

Lifestyle factors such as diet and exercise contribute largely to obesity and other cardio-metabolic disorders. Several studies have shown that a low carbohydrate diet can decrease *EreC* group proportions [29,30]. A decreased *Firmicutes* to *Bacteroidetes* ratio was found in an intervention with a calorie-restricted diet [31]. Our result showed that the *EreC* group was negatively correlated with carbohydrate intake but positively with fat intake. No association was found between the *EreC* group and fiber intake. Earlier studies have indicated that a dietary-resistant (or “non-digestive”) carbohydrate—through microbial conversion to short-chain fatty acids (SCFA)—is less effective than the equivalent amount of sugar absorbing directly in the small intestine for harvesting the energy [29]. SCFAs serve as energy substrates for the epithelial cells of the gut and provide part of the dietary energy [29,32], while other studies have shown that microbial pathways which generate SCFAs were enriched in metagenomics studies of obese subjects, and levels of SCFAs were elevated in overweight or obese people and animal models [25,33]. Hence the changes in gut microbiota composition that result from different dietary intakes are still inclusive.

The underlying mechanisms by which cardiorespiratory fitness might be associated with microbiota are yet to be fully understood. A recent study by Estaki et al. [10] showed that cardiorespiratory fitness is associated with increased gut microbial diversity. The association between cardiorespiratory fitness and certain bacteria in our study was confounded by adiposity, which raised the question of whether cardiorespiratory fitness plays a role in altering gut microbiota. One of the possible links between cardiorespiratory fitness and certain bacteria may be that physical activity decreases total colonic transit time. Long-term regular physical activity (resulting in high cardiorespiratory fitness) has a positive effect on both constipation indices and rectosigmoid transit time [34,35]. An earlier study has showed that a short gastrointestinal tract (GIT) resulting from gastric bypass enriched the gut microbiota of phylum level Bacteroidetes, Verrucomicrobia, and Proteobacteria after the surgery [36]. It is possible that the speed of colonic transit might be higher in those subjects who had higher VO_2max_. Furthermore, physically active individuals are more likely to follow a healthy lifestyle and to be more exposed to their environmental biosphere, and this may also contribute to a different composition of microbiota. Simultaneously, adaptation and the acute effect of endurance training can lead to changes in the GIT, such as increased transit and absorptive capacity, tissue hypoxia, and decreased blood flow [37,38]. These and other potential mechanisms, such as changes in gut pH, may create an environmental setting which limits the growth of the *EreC* group. On the other hand, controlling for fat%, the association between the *EreC* group and VO_2max_ disappeared and fat% contributes the most in the regression model. Hence, whether exercise or fitness level has significant impact on the time of chyme staying in the intestinal tract, altering the composition of gut microbiota, deserves further study.

An earlier study has reported that the gut microbiome contributes to a substantial proportion of the variation in blood lipids [28,39]. Gut microbiota have been linked with lipid metabolism through their role in bile acid metabolism [40]. Fu et al. used 16s rRNA gene sequencing and found that members of family *Ruminococcaceae* (OTU 175962, 1703711, 295743, 178385) were negatively correlated with triglycerides, but certain members of the genus *Eubacterium* (OTU 49837) positively correlated with triglycerides [28]. However, Karlsson et al. used shotgun sequencing to find that the *Clostridium* species, *Ruminococcus* species and *Eubacterium rectale* were correlated positively with triglycerides and leptin, and negatively with HDL [41]. We found that a high proportion of *EreC* was associated with high triglycerides, but low HDL. Controlling for fat%, the significant associations between the *EreC* group and triglycerides remained, but disappeared between the *EreC* group and HDL. Since the *EreC* group includes members of *Clostridium oroticum*, *Clostridium nexile*, *Ruminococcus hansenii*, *Ruminococcus productus*, and *Eubacterium rectale*, it indicates the complexity of the bacterial in relation to the clinical outcomes. Nerveless, this could partly explain why different associations were reported for the *EreC* group with triglycerides and HDL.

Our study had some limitations. First, the study used a cross-sectional design and therefore no cause and effect relationship could be identified. Second, the method we used to assess gut microbiota was based on the detection of conserved regions of the 16S ribosomal RNA gene [42]. Though the method we used can detect the specific groups and clusters of gut microbiota [43,44,45,46], it cannot reach the deeper taxonomical levels such as species and strains, which might be important in the assessment of the functional role of intestinal microbiota. Thus, a more detailed structural and functional analysis of gut microbiota based on, for example, next generation sequencing methods in a larger scale of the population is required in order to explore the entire gut microbiota community [47]. One other limitation is that most of our participants were overweight or obese with relatively low fitness levels, this may have resulted in the underestimation of the association between VO_2max_ and bacteria. Finally, only premenopausal women were included in this study, thus the effect of estrogen on the gut microbiota [11] cannot be verified. Nerveless, our results provide new information that there is a moderate association between physical fitness and certain gut microbiota in premenopausal women.

## 5. Conclusions

In conclusion, a low proportion of *EreC* in the gut is associated with high aerobic fitness in women, independent of age and dietary intakes. However, the relationship between VO_2max_ and *EreC* is confounded by body fatness. In addition, the *EreC* group was positively associated with triglycerides. Our study indicates that further studies are needed to verify the relationships between gut microbiota, lipids and cardiorespiratory fitness.

## Figures and Tables

**Table 1:** Physical characteristics, background and gut microbiota composition in the low, moderate and high cardiorespiratory fitness groups (means with 95% confidence intervals are given and contrast estimates (*p*-values) by multivariable comparison with Sidak to localize the significant differences between the groups).

Variables	Low VO_2max_ Mean 95% CI	Moderate VO_2max_ Mean 95% CI	High VO_2max_ Mean 95% CI	ANOVA (*p*-Value)		
Number	24	23	24	**L-M**	**L-H**	**M-H**
Age (years)	40.4 (36.9–44.0)	39.7 (35.5–43.8)	30.6 (25.6–35.6)	0.991	0.003	0.009
Height (cm)	166.1 (164.0–168.3)	166.7 (163.9–168.9)	164.0 (161.7–166.3)	0.984	0.490	0.307
Weight (kg)	87.3 (83.2–91.5)	77.6 (73.0–82.1)	66.1 (62.1–70.1)	0.004	<0.001	0.001
BMI	31.7 (30.2–33.1)	27.9 (26.7–29.1)	24.6 (23.0–26.2)	0.001	<0.001	0.004
Fat%	40.6 (38.1–43.0)	35.5 (33.2–37.8)	28.0 (25.0–31.0)	0.018	<0.001	<0.001
PA (time/week)	1.39 (0.95–1.83)	1.80 (1.22–2.38)	3.44 (2.61–4.27)	0.718	<0.001	0.001
PA (h/week)	1.17 (0.69–1.64)	2.11 (1.27–2.95)	3.69 (2.82–4.56)	0.195	<0.001	0.009
Fiber (g)	20.4 (17.4–23.5)	24.1 (20.6–27.6)	21.0 (17.3–24.6)	0.323	0.995	0.450
Carbohydrate (E%)	47.1 (43.7–50.5)	45.6 (41.7–49.5)	44.7 (41.5–48.0)	0.901	0.698	0.979
Protein (E%)	18.4 (17.0–19.8)	18.0 (17.0–19.0)	18.0 (16.9–19.1)	0.937	0.957	1.000
Fat (E%)	32.8 (30.3–35.4)	34.1 (30.9–37.2)	35.2 (32.5–38.0)	0.887	0.508	0.910
Alcohol (E%)	1.73 (−54–3.99)	2.38 (0.45–4.31)	0.63 (−37–1.62)	0.958	0.955	0.769
TG (mmol/L)	1.56 (1.17–1.95)	1.22 (1.02–1.42)	1.01 (0.82–1.20)	0.608	0.041	0.434
Cholestol (mmol/L)	5.06 (4.55–5.56)	5.03 (4.68–5.38)	4.44 (4.14–4.74)	0.999	0.072	0.098
HDL (mmol/L)	1.42 (1.29–1.55)	1.59 (1.47–1.72)	1.57 (1.42–1.73)	0.198	0.279	0.996
Leptin (ng/mL)	56.8 (45.8–67.8)	41.7 (34.5–48.8)	26.9 (18.2–35.6)	0.054	<0.001	0.062

BMI = body mass index; Fat% = percent body fat; PA = physical activity; E% = energy yield; TG = triglycerides; HDL = High-density lipoproteins.

**Table 2:** Linear regression of *EreC* with VO_2max_, fat%, leptin, HDL and TG.

	*EreC*		*Erec* Adjusted for Age and Energy Yield Macronutrients Intakes of Carbohydrate, Protein, Fat and Alcohol		*EreC* Adjusted for Age, Fat% and Energy Yield Macronutrients Intakes of Carbohydrate, Protein, Fat and Alcohol	
	*r*	*p*	*r*	*p*	*r*	*p*
VO_2max_	−0.309	0.010	−0.339	0.006	−0.113	0.369
Fat%	0.382	0.002	0.364	0.005		
Leptin	0.424	0.001	0.390	0.003	0.178	0.208
HDL	−0.260	0.028	−0.313	0.010	−0.233	0.124
TG	0.390	0.002	0.393	0.003	0.300	0.060

Fat% = percent body fat; HDL = High-density lipoproteins; TG = triglycerides; *EreC* = *Eubacterium rectale-Clostridium coccoides*; *p* value was adjusted by multiple comparisons in FDR (false discovery rate).

**Table 3:** The regression weights of multivariable associations.

	Y	VO_2max_	Leptin	HDL	TG
X					
*EreC*		−0.0711	0.1206	−0.2494	0.3277
Age		−0.3369	−0.0917	0.1579	0.0618
Fat%		−0.6163	0.7887	−0.2130	0.3008
Carbohydrates (E%)		−0.2886	−0.2693	0.2631	−0.1922
Protein (E%)		−0.0680	−0.1145	−0.0252	−0.0104
Fat (E%)		−0.0520	−0.1298	0.3869	−0.1923
Alcohol (E%)		−0.1851	−0.1049	0.1990	−0.3277

*EreC* = *Eubacterium rectale-Clostridium coccoides*; HDL = High-density lipoproteins; TG = triglycerides; Fat% = percent body fat; E% = energy yield.

**Table 4:** The regression weights of multivariable associations.

	Y	VO_2max_	Fat%	Leptin	HDL	TG
X						
*Bif*		0.0348	0.1092	0.0892	−0.0679	0.1539
*Bacto*		0.1536	−0.1479	−0.0939	0.2540	−0.1184
*F.p*		0.0113	0.0929	−0.0080	0.1721	−0.1377
*Ent*		−0.0723	−0.0129	−0.0016	0.1995	−0.0139
*EreC*		−0.2568	0.3276	0.3942	−0.3129	0.3894

Bif = Bifidobacterium, F.p = Faecalibacterim prausnitzii, Bacto = Bacteroides, Ent = Enterobacteria, EreC = Eubacterium rectale-Clostridium coccoides; Fat% = percent body fat; HDL = High-density lipoproteins; TG = triglycerides.
